# Supplementary material for: Epidemiology of Clostridioides difficile in South Africa
Source: PLoS One. 2021 Nov 23;16(11):e0259771. doi: 10.1371/journal.pone.0259771 (PMC8610264; doi:10.1371/journal.pone.0259771)
Supplement: Attachment — Submitted filename: CD Manuscript Review comments-PONE-D-21-03916.docx [file pone.0259771.s002.docx]

Manuscript title: Epidemiology of Clostridioides difficile in South Africa

Manuscript number: PONE-D-21-03916

Review comments:

1. Abstract (Pg 9/iii)- This is well written and summarized. However, the method (study design) is not clearly stated; whether it was a retrospective, prospective or cohort study. The results section also talks of 14023 samples were tested for CD raising concerns on whether the authors did the actual testing or rather obtained data on the tested cases/samples. The conclusion also talks about the incidence being lower compared to high income countries which may have been worth be compared with data from developing countries as well.
2. Introduction: Well written and adequate
3. Methods: The study design is missing or not stated to support the data generated.
4. Results: Well-articulated and tabulated appropriately in tables 1-5
5. Discussion: Clearly stated based on the data generated and previous data
6. Conclusion: Sufficient according to the data obtained.
