# Supplementary material for: Epidemiology of Clostridioides difficile in South Africa
Source: PLoS One. 2021 Nov 23;16(11):e0259771. doi: 10.1371/journal.pone.0259771 (PMC8610264; doi:10.1371/journal.pone.0259771)
Supplement: Attachment — Submitted filename: CDI_Response_V3_2.7.21.docx [file pone.0259771.s003.docx]

| This work is about the incidence of clostridioides difficile infection (CDI) in South Africa (SA). Current knowledge, mostly derived from high income countries, suggests that clostridioides difficile (CD) is a major cause of healthcare associated diarrhea and it is increasingly present in the community. This manuscript contributes to raise clinical awareness of its existence in SA and, I dare say, to the all African continent. | Thank you for the positive response. |
| --- | --- |
|  |  |
| To estimate the incidence, as numerator, the authors took the [newly?] number of cases diagnosed through the National Health Laboratory Service (NHLS) between 1 July 2016 and 30 June 2017. According to the authors the NHLS has a national database of all processed laboratory results in the South Africa public sector [the public health sector which covers 84% of South Africa population]. For the denominator they took the overall mid-year population of South Africa in 2016. Therefore, the CDI incidence is underestimated for both underestimated numerator and large denominator, but I agree with them that it is useful estimate. | We only included newly diagnosed cases of CDI to estimate the incidence of C. Diff in South Africa. All cases of recurrence or other duplicate cases were excluded.  For the denominator we took the mid-year population estimates for each province and adjusted the population estimate for the percentage of the population covered by the private sector. This adjustment was done to obtain a more accurate estimate of CDI incidence for each province and nationally. (see line 182, page 4) |
| In addition to the incidence estimation, the authors produce some measure of association using as exposures (the call risk factors) age, gender, level of care and ICU/HC, and outcomes the state of positive or negative to diagnosis of CDI. Although the negatives here are not good controls it is OK to this exercise. | Thank you, we agree that a better control would be patients without any diarrhoeal disease per se but rather a random population sample. Unfortunately, our database only included patients with diarrhoeal disease. |
| Few issues: In the next version please add line numbering. That helps to track and indicate the place of an issue. | Line numbers have been inserted. |
| I. Introduction is adequate and quite instructive. | Thank you |
| II. Methods: Why the study of risk factors ignore incidence rates? The authors proceed as if they built a case-control with OR estimation. I guess you can obtain population per age and gender. That could be used on a Poisson regression which gives IRR/RR. Just a clarification. | Thank you for this comment. We have re-worked the entire analysis for the risk factor section. We agree that is more appropriate to express risk in terms of IRR and therefore re-analysed the data utilizing Poisson regression |
| 1. Somewhere in the first paragraph there is an indication that a STROBE form was filled. I did not find the attached form. Please add this in the supplements. | Thank you, this has been included. |
| 2. Data management and analysis - For the mid-year population conceptually it represents the number of people living in a specific geographic area on 1st July (others would say 30th of June). That is too simplistic. It is more useful to think of it as the total of the fractions year that each individual in South Africa lived. Eg: some lived the full year so their fraction is 1; others just the first quarter so 0.25; and others up to 3rd quarter so 0.75 and so on. So it is a person-years contribution to one year. So, why the authors here decided to mid-year population of 2016 and the numerator covers 2 years? The mid-year population between 1st July 2016 and 30th June 2017 is something close to 1st January of 2017. Just a clarification please. | Thank you for your comment. Unfortunately, we did not have access to granular migration data to allow us to estimate person-years.  We utilized mid-year population estimates for 2016 as this represented the ‘starting time’ of our study and the potential population at risk. Our study period was for one year only (numerator consisted of all cases identified between 1 July 2016 and 30 June 2017)  It is true that utilizing the 2016 mid-year estimate rather than the 2017 mid-year estimate may impact our results. However, we do not believe that it would have a major impact: for example, the province with the most immigration in South Africa (Gauteng) population increased by 5.46% from 2016 – 2017 and the overall population increased by 1.08% (see below tables of provincial estimates for 2016 and 2017). Further, the South African government (StatSA) only releases mid-year estimates, no estimates were released for 1 January 2017 |
| - State in the methods how the CID incidence confidence intervals were estimated. | We’ve stated this in the text (line 225), we used Poisson regression – our STATA coding was for example:  *ci means positive, poisson exposure(population)* |
| - The statement starting as “The NHLS only test specimens for CD that conform to the shape of the container”. This is unclear and confusing. Please clarify. | Thank you, we have clarified this statement: only unformed (Bristol stool class 5 – 7) specimens are tested for C Diff |
| - Figure 1 is important but it needs changes to 1) to separate clearly exclusions and 2) distinguish sample count from patient counts. Otherwise we will be confused on the calculations as one of the reviewers rightly asks. A sort of suggestion is attached as picture [pardon me for lack of technological sophistication, see the photo of a drawing attached]. | Thank you, we agree that this figure required additional clarification. The figure has been extensively revised. |
| - This paragraph related to figure 1 has a lot of information that belongs to results section. Please review. | Thank you, we have moved this paragraph from the methods to the results section |
| - Be careful when interpreting odds-ratios as risk-ratios as in, for example, “receiving care in a specialized TB hospital resulted in a more than 11-fold increase in the risk of CDI”. This is incorrect. An example: the proportions of positivity among males and females are 19.12% and 18.28%, respectively. So the is [19.12/(100 – 19.12)]/[18.28/(100 – 18.28)] = 1.06 which is not a ratio of proportion 19.12/18.28 = 1.05. The difference between these two gets worse for higher OR. | Thank you for this valuable comment, we’ve amended the document and the analysis |
| III. Results 1. Table 1. * The percentages cause confusion. I propose these changes - Please add a row of totals - For the 3 columns ‘n(%)’, ‘CDI’, and ‘non-CID’ make them the percentages be in columns - Add one column named ‘CD positivity rate’ so the current percentage calculated in CDI would move to a column dedicated for row percentage. - Why we need p-values here? Those p-values inform less than the ones on table 2. | Apologies, but we’re not totally clear on what the reviewer is requiring from us here:  The first column provides the total sample for each category (N), we’ve removed the percentages from this column  The % in column 2 ‘CDI n (%)’ has been changed. Previously it gave the positivity rate for each category, it now reflects the % of subjects in each category with the total number of subjects in the denominator.  We agree that the p-values in table 2 are more informative. We included the p-values as they guided us in assessing which variables (e.g. age) overall may have a significant association with the outcome of interest (i.e. CDI), in table two we then looked at which category (e.g. age 31 – 41 vs reference age category 1 – 30) may have a significant association with CDI. We would, if you agree, elect to keep the p-values in table 1. |
| * One of the reviewers complains about the numbers. Please re-verify the calculations. - Eg: The positivity frequency for females 974/5,329 = 18.28% that is OK. But for males should be 756/3,953 = 19.12%. * Why province is not here? | Thank you, we’ve rechecked the values in the table and amended any errors  We did not included provinces in the table as we, for the purposes of this analysis, did not view ‘province’ as a risk factor for CDI |
| 2. Table 2 – * Why province is not here? | Table 2 assessed risk factors potentially associated with CDI. We did not consider ‘province’ as a risk factor for CDI |
| 3. Table 3 needs similar changes as table 1. | As per our response for table 1 above |
| 4. Table 4 – please what is the unity of measurement of the incidence. Remove the positivity rate. That is fine on table one. Three columns are missing. One for the population and the numerators. That will be important in a future meta-analysis. Add a row for the total. This is important to understand the 0.101 described in the results. | Unit of measure is “per 100 000 admissions”, this has been included in the table  Positivity rate has been removed  We’ve added a column for the total admissions and total number of CDI cases by province |
| 5. Table 5 – the same comments as for table 4.be careful with abbreviation of CD. | We amended the table accordingly |
| 6. The first paragraph of results ends with something as “On univariate analysis only level of patient care was statistically significantly associated with CDI (p < 0.001) (table 1)” Please do not use p-values as evidence of associations. For that, first describe the association measure. | Thank you, we agree and have elected to remove the statement |
| 7. If the p-value is below the 0.001 please write “p-value < 0.001” not “p = 0.000” as in “81% (OR 1.81 CI95% 1.51 – 2.17, p = 0.000)”. | This has been edited accordingly |
| 8. All figures are numbered figure 1. Please correct this. | This has been corrected |
| 9. I am not sure about the seasonality based just with one year of data. Please note this limitation in your discussion | This has been addressed in line 420 - 421 |

| Journal Requirements:  Please review your reference list to ensure that it is complete and correct. If you have cited papers that have been retracted, please include the rationale for doing so in the manuscript text, or remove these references and replace them with relevant current references. Any changes to the reference list should be mentioned in the rebuttal letter that accompanies your revised manuscript. If you need to cite a retracted article, indicate the article’s retracted status in the References list and also include a citation and full reference for the retraction notice.  When submitting your revision, we need you to address these additional requirements. | Thank you, references have been reviewed |
| --- | --- |
| 1. Please ensure that your manuscript meets PLOS ONE's style requirements, including those for file naming. The PLOS ONE style templates can be found at  and  <https://journals.plos.org/plosone/s/file?id=ba62/PLOS>  One_formatting_sample_title_authors_affiliations.pdf | Document has been edited to align with PLOS ONE style |
| 2. Thank you for providing the date(s) when patient medical information was initially recorded. Please also include the date(s) on which your research team accessed the databases/records to obtain the retrospective data used in your study. | We’ve added the following “Data were accessed on 10 July 2020” see line 144 |
| 3. To comply with PLOS ONE submission guidelines, in your Methods section, please provide additional information regarding your statistical analyses. For more information on PLOS ONE's expectations for statistical reporting, please see <https://journals.plos.org/plosone/s/submission-guidelines.#loc-statistical-reporting>. | Additional information has been provided |
| 4. In your Methods section, please provide additional information about the participant recruitment method and the demographic details of your participants. Please ensure you have provided sufficient details to replicate the analyses such as: a) a description in the methods section of any inclusion/exclusion criteria that were applied to participant selection, b) a statement as to whether your sample can be considered representative of a larger population. | We only had age and sex available for demographic information  This has been described in the methods section and Figure 1  We’ve added the following statement (Line 178/179)….our sample can be considered representative of the South African public sector |
| 5. Thank you for stating in your ethics statement "The protocol for this study was reviewed by the Human Research Ethics Committee (Medical) at the University of the Witwatersrand, Johannesburg and clearance was obtained (Ref: M200114) prior to commencing the study. This was a retrospective study utilizing secondary data sources. Prior to data analysis all unique identifiers were delinked to ensure anonymity." In your ethics statement, please clarify:  - whether the ethics committee specifically approved this study.  - whether consent was obtained  - whether consent was informed  - what type of consent you obtained (for instance, written or verbal, and if verbal, how it was documented and witnessed).  - if your study included minors, state whether you obtained consent from parents or guardians.  - if the need for consent was waived by the ethics committee, please include this information. | We’ve edited our ethics section to include the following:  Approval for this specific study was obtained  Consent/permission was obtained from the NHLS to access the required data  Our study included data derived from minors over the age of 1 year |
| 6. We note that you have indicated that data from this study are available upon request. PLOS only allows data to be available upon request if there are legal or ethical restrictions on sharing data publicly. For information on unacceptable data access restrictions, please see <http://journals.plos.org/plosone/s/data-availability#loc-unacceptable-data-access-restrictions>.   In your revised cover letter, please address the following prompts:     a) If there are ethical or legal restrictions on sharing a de-identified data set, please explain them in detail (e.g., data contain potentially identifying or sensitive patient information) and who has imposed them (e.g., an ethics committee). Please also provide contact information for a data access committee, ethics committee, or other institutional body to which data requests may be sent.   b) If there are no restrictions, please upload the minimal anonymized data set necessary to replicate your study findings as either Supporting Information files or to a stable, public repository and provide us with the relevant URLs, DOIs, or accession numbers. Please see <http://www.bmj.com/content/340/bmj.c181.long> for guidelines on how to de-identify and prepare clinical data for publication. For a list of acceptable repositories, please see <http://journals.plos.org/plosone/s/data-availability#loc-recommended-repositories>.    We will update your Data Availability statement on your behalf to reflect the information you provide. | Upon closer consideration we are of the opinion that all relevant data are reported in the manuscript. |
| 7. Please amend either the abstract on the online submission form (via Edit Submission) or the abstract in the manuscript so that they are identical. | Thank you, on-line abstract has been amended. |
| 8. We note that Figure 3 in your submission contain map images which may be copyrighted. All PLOS content is published under the Creative Commons Attribution License (CC BY 4.0), which means that the manuscript, images, and Supporting Information files will be freely available online, and any third party is permitted to access, download, copy, distribute, and use these materials in any way, even commercially, with proper attribution. For these reasons, we cannot publish previously copyrighted maps or satellite images created using proprietary data, such as Google software (Google Maps, Street View, and Earth). For more information, see our copyright guidelines: <http://journals.plos.org/plosone/s/licenses-and-copyright>.    We require you to either (1) present written permission from the copyright holder to publish these figures specifically under the CC BY 4.0 license, or (2) remove the figures from your submission:  8.1.    You may seek permission from the original copyright holder of Figure 1 to publish the content specifically under the CC BY 4.0 license.    We recommend that you contact the original copyright holder with the Content Permission Form (<http://journals.plos.org/plosone/s/file?id=7c09/content-permission-form.pdf>) and the following text:  “I request permission for the open-access journal PLOS ONE to publish XXX under the Creative Commons Attribution License (CCAL) CC BY 4.0 (<http://creativecommons.org/licenses/by/4.0/>). Please be aware that this license allows unrestricted use and distribution, even commercially, by third parties. Please reply and provide explicit written permission to publish XXX under a CC BY license and complete the attached form.”    Please upload the completed Content Permission Form or other proof of granted permissions as an "Other" file with your submission.    In the figure caption of the copyrighted figure, please include the following text: “Reprinted from [ref] under a CC BY license, with permission from [name of publisher], original copyright [original copyright year].”  8.2.    If you are unable to obtain permission from the original copyright holder to publish these figures under the CC BY 4.0 license or if the copyright holder’s requirements are incompatible with the CC BY 4.0 license, please either i) remove the figure or ii) supply a replacement figure that complies with the CC BY 4.0 license. Please check copyright information on all replacement figures and update the figure caption with source information. If applicable, please specify in the figure caption text when a figure is similar but not identical to the original image and is therefore for illustrative purposes only.  The following resources for replacing copyrighted map figures may be helpful:    USGS National Map Viewer (public domain): <http://viewer.nationalmap.gov/viewer/>  The Gateway to Astronaut Photography of Earth (public domain): <http://eol.jsc.nasa.gov/sseop/clickmap/>  Maps at the CIA (public domain): <https://www.cia.gov/library/publications/the-world-factbook/index.html> and <https://www.cia.gov/library/publications/cia-maps-publications/index.html>  NASA Earth Observatory (public domain): <http://earthobservatory.nasa.gov/>  Landsat: <http://landsat.visibleearth.nasa.gov/>  USGS EROS (Earth Resources Observatory and Science (EROS) Center) (public domain): [http://eros.usgs.gov/#](http://eros.usgs.gov/)  Natural Earth (public domain): <http://www.naturalearthdata.com/> | These images were generated by the authors  Shape files were obtained from an open source GADM (https://gadm.org/data.html), with freely available data for academic and non-commercial use |
| 9. Please include a caption for figures 2 and 3. | We’ve included captions |

| Manuscript title: Epidemiology of Clostridioides difficile in South Africa  Manuscript number: PONE-D-21-03916  Review comments:   1. Abstract (Pg 9/iii)- This is well written and summarized. However, the method (study design) is not clearly stated; whether it was a retrospective, prospective or cohort study. The results section also talks of 14023 samples were tested for CD raising concerns on whether the authors did the actual testing or rather obtained data on the tested cases/samples. The conclusion also talks about the incidence being lower compared to high income countries which may have been worth be compared with data from developing countries as well. | We have amended the methods section to clearly state this is a retrospective cohort study  The authors did not conduct the actual testing. All stool specimens were tested by the National Health Laboratory Service (NHLS) and data for analysis were obtained from the NHLS  There are very limited data on developing countries, therefore we primarily discussed finding from high income countries |
| --- | --- |
| 2. Conclusion: Sufficient according to the data obtained. | Thank you |
| 3. Methods: The study design is missing or not stated to support the data generated. | This has been amended. |
| 4. Results: Well-articulated and tabulated appropriately in tables 1-5 | Thank you, some edits have been made to the tables based on comments from other reviewer(s) |
| 5. Discussion: Clearly stated based on the data generated and previous data | Thank you for the comment |
| 6. Introduction: Well written and adequate | Thank you for the comment |
